# The short-term impact of methylprednisolone on patient-reported sleep in patients with advanced cancer in a randomized, placebo-controlled, double-blind trial

**DOI:** 10.1007/s00520-020-05693-6

**Published:** 2020-08-27

**Authors:** Gunnhild Jakobsen, Morten Engstrøm, Marianne Jensen Hjermstad, Jan Henrik Rosland, Nina Aass, Eva Albert, Stein Kaasa, Peter Fayers, Pål Klepstad, Ørnulf Paulsen

**Affiliations:** 1 Department of Clinical and Molecular Medicine, Faculty of Medicine and Health Sciences, NTNU Norwegian University of Science and Technology, Postbox 8905, N-7491 Trondheim, Norway; 2 Cancer Clinic, St. Olavs Hospital, Trondheim University Hospital, Trondheim, Norway; 3 Department of Neuromedicine and Movement Science, NTNU - Norwegian University of Science and Technology, Trondheim, Norway; 4 Department of Neurology and Clinical Neurophysiology, St. Olavs Hospital, Trondheim University Hospital, Trondheim, Norway; 5 Regional Advisory Unit for Palliative Care, Department of Oncology, Oslo University Hospital, Oslo, Norway; 6 European Palliative Care Research Centre, Department of Oncology, Oslo University Hospital, Oslo, Norway; 7 Institute of Clinical Medicine, University of Oslo, Oslo, Norway; 8 Department of Clinical Medicine, University of Bergen, Bergen, Norway; 9 Sunniva Centre for Palliative Care, Haraldsplass Deaconess Hospital Bergen, Bergen, Norway; 10 Institute of Clinical Medicine, Faculty of Medicine, University of Oslo, Oslo, Norway; 11 Sørlandet Hospital Kristiansand, Kristiansand, Norway; 12 Division of Applied Health Sciences, University of Aberdeen, Aberdeen, UK; 13 Department of Anaesthesiology and Intensive Care Medicine, St. Olavs Hospital, Trondheim University Hospital, Trondheim, Norway; 14 Department of Circulation and Medical Imaging, Faculty of Medicine and Health Sciences, NTNU - Norwegian University of Science and Technology, Trondheim, Norway; 15 Telemark Hospital Trust, Skien, Norway

**Keywords:** Corticosteroids, Sleep, Advanced cancer, Randomized controlled trial

## Abstract

**Purpose:**

Although corticosteroids are frequently used in patients with advanced cancer, few studies have examined the impact of these drugs on patient-reported sleep. We aimed to examine the short-term impact of methylprednisolone on patient-reported sleep in patients with advanced cancer.

**Methods:**

Patient-reported sleep was a predefined secondary outcome in a prospective, randomized, placebo-controlled, double-blind trial that evaluated the analgesic efficacy of corticosteroids in advanced cancer patients (18+), using opioids, and having pain ≥ 4 past 24 h (NRS 0–10). Patients were randomized to the methylprednisolone group with methylprednisolone 16 mg × 2/day or placebo for 7 days. The EORTC QLQ-C30 (0–100) and the Pittsburgh Sleep Quality Index questionnaire (PSQI) (0–21) were used to assess the impact of corticosteroids on sleep at baseline and at day 7.

**Results:**

Fifty patients were randomized of which 25 were analyzed in the intervention group and 22 in the control group. Mean age was 64 years, mean Karnofsky performance status was 67 (SD 13.3), 51% were female, and the mean oral daily morphine equivalent dose was 223 mg (SD 222.77). Mean QLQ-C30 sleep score at baseline was 29.0 (SD 36.7) in the methylprednisolone group and 24.2 (SD 27.6) in the placebo group. At day 7, there was no difference between the groups on QLQ-C30 sleep score (methylprednisolone 20.3 (SD 32.9); placebo 28.8 (SD 33.0), *p* = 0.173). PSQI showed similar results.

**Conclusions:**

Methylprednisolone 16 mg twice daily for 7 days had no impact on patient-reported sleep in this cohort of patients with advanced cancer.

**Trial registration:**

Clinical trial information NCT00676936 (13.05.2008)

## Background

Patients with advanced cancer often report poor sleep quality with a prevalence that varies from 40 to 96% across studies [1–6]. The cancer disease and the cancer treatments are factors that probably contribute to sleep disturbances [7]. Treatment with corticosteroids may be a precipitating factor involved in the development of insomnia in patients with cancer [8–11]. Corticosteroids are associated with a variety of adverse effects in which sleep disturbance is one of the short-term effects [12–15]. These drugs are frequently administered to patients with advanced cancer [16–21], but usage varies between countries. For instance, a cross-sectional study of European cancer patients using opioids showed that 34% in Germany, 49% in Norway, and 72% of patients in Italy were using corticosteroids [22]. The indications for corticosteroid use in patients with advanced cancer are wide, including alleviation of distressing symptoms such as pain, anorexia, fatigue, and dyspnea as well as improving quality of life and sense of well-being [16, 17, 20, 21]. Some patients also use corticosteroids as a part of anti-cancer therapy or for other medical conditions.

Few studies have examined the impact of corticosteroids on patient-reported sleep in patients with advanced cancer. Mercadante et al. demonstrated an inverse relation between the use of corticosteroids and sleep assessed by the Athens Insomnia Scale in patients with advanced cancer [23]. However, due to the cross-sectional design, causality between corticosteroids and sleep quality could not be assessed. Second, in a prospective survey on the use of dexamethasone in in and outpatients with advanced malignant disease, Hardy et al. demonstrated that mild sleep disturbance was reported by 17% of the patients after start of corticosteroid treatment [24]. In another study, Hatano et al. demonstrated that 19% of patients with advanced cancer experienced sleep disturbance 1 week after start of dexamethasone given for anorexia [25]. In the latter study, sleep was rated by healthcare workers according to the National Cancer Institute’s Common Toxicity Criteria for Adverse Events Likert scales. However, as sleep disturbance was not assessed by patient-reported outcome measures, it is likely underestimated. Furthermore, none of these studies used standardized doses of corticosteroids nor did they include a control group. Finally, Yennurajalingam et al. performed a randomized, double-blind, placebo-controlled study on the use of dexamethasone 4 mg orally twice daily for 14 days for cancer-related fatigue in patients with advanced cancer. They found no difference in sleep using a numeric scale (NRS), (0, no symptoms; 10, worst possible severity) between dexamethasone and placebo at day 15 [26].

To summarize, few studies have examined the impact of standardized doses of corticosteroids on sleep by a longitudinal randomized controlled design in patients with advanced cancer. Given the high prevalence of both sleep disturbances and corticosteroid use in patients with advanced cancer, it is essential to know whether corticosteroids aggravate sleep in these patients. The aim of this study was to investigate patient-reported sleep, a predefined secondary outcome in the randomized Corticosteroids for Cancer Pain Trial (NCT00676936) [27].

## Methods

### Overall design and participants

The randomized Corticosteroid for Cancer Pain Trial [27] investigated the analgesic effect of methylprednisolone on pain, with patient-reported sleep as a predefined secondary outcome in a prospective, randomized, placebo-controlled, double-blind design [27]. Eligible patients in the randomized controlled trial had verified malignant disease, aged 18 years or above, self-reported average pain ≥ 4 last 24 h (assessed on a Numerical Rating Scale (NRS) 0–10), used opioids for moderate or severe cancer pain, and had an expected length of survival of more than 4 weeks. Exclusion criteria were severe pain, defined as 8 or above on the NRS last 24 h; regular corticosteroid use; initiated radiotherapy or systematic cancer treatment in the past 4 weeks; diagnosed with diabetes mellitus, peptic ulcer disease, spinal cord compression, or being in need of bone surgery; and ongoing treatment with non-steroidal anti-inflammatory drugs. Patients with obvious cognitive impairment as judged by the treating physician according to standard criteria (e.g., confusion, disorientation, low attention span and incoherent speech) were also not eligible [27]. Five palliative care and outpatient oncology services in Norway participated in the study [27].

### Study treatment

Patients were randomized to methylprednisolone 16 mg or placebo twice daily for 7 days. The study drug was administered in the morning and before 6 p.m. in the afternoon. Patients and members of the research team were all blinded to treatment assignment throughout the study. Details of the study procedures are described in the original article [27].

### Outcome measures

#### Sleep

Sleep, a secondary outcome predefined in the protocol [27], was assessed at baseline and day 7 using the European Organization for Research and Treatment of Cancer Quality of Life Questionnaire Core 30 (QLQ-C30) [28]. The QLQ-C30 includes a single question assessing sleep difficulty: “(During the past week) Have you had trouble sleeping?” The QLQ-C30 sleep item has four response categories from “not at all” to “very much”. The raw scores are transformed linearly to a scale from 0 to 100, i.e., QLQ-C30 sleep score, where a high score represents more sleeping trouble [28]. A mean difference of 10–20 points is described as a moderate change that is perceptible to patients [29]. Thus, a difference of 15 points on the QLQ-C30 sleep score was considered clinically significant in this trial.

Sleep quality was also assessed by the Pittsburgh Sleep Quality Index questionnaire (PSQI) [30]. The PSQI is a 19-item self-report questionnaire that assesses sleep quality. The recall period is 1 month. The questionnaire comprises seven domains: subjective sleep quality, sleep latency, sleep duration, normal sleep efficiency, sleep disturbances, the application of sleep medicine, and daytime dysfunction [30]. Each domain has a response score ranging from 0 to 3, with lower scores indicating better sleep quality. The seven component scores are summed to yield a PSQI total global score (0–21). PSQI total global scores ˃ 5 indicate poor sleep [4, 30].

Cognitive function was assessed pre- and posttreatment by the Mini Mental State Exam (MMSE). The MMSE has 20 items and a sum score of 30 points [31]. Performance status was rated by the Karnofsky performance status (KPS) [32]. Patients reported their daily analgesic consumption, and opioid dosages were converted to oral morphine equivalents [33].

### Statistical analysis

Power calculations were presented in the original article and were based on the primary outcome of average pain intensity measured at day 7 (on an NRS 0–10 (0 = no pain, 10 = worst imaginable pain) [27]. The estimated sample size was 22 evaluable patients in each group.

Demographic variables are reported as means with standard deviation (SD) or frequencies. The main sleep outcome was the QLQ-C30 sleep score day 7 after treatment. Difference between the intervention group and the control group in QLQ-C30 sleep score on day 7 was assessed using a general linear model (ANCOVA) including baseline values as a covariate in the model. We also calculated the change score for each patient by subtracting the baseline sleep score (day 0) from the score at end of treatment (day 7).

Differences between the two groups were also assessed in explorative analyses using the global PSQI score and each of the PSQI dimensions as the dependent variable in the ANCOVA model, i.e., subjective sleep quality, sleep latency, sleep duration, sleep efficiency, sleep disturbances, sleep medication, and dysfunction (0–3), and the patient-reported sleep onset latency in minutes. The baseline value was in each case used as a covariate in the model.

There was one missing item in the PSQI-sleep disturbance scale in the PSQI. This was imputed by the last observation carried forward. One patient had a missing value in the QLQ-C30 sleep item at baseline, and one patient had a missing value at day 7; no imputation method was used, as these were single items.

A two-sided *P* value of ˂ 0.05 was considered statistically significant. *P* values were not adjusted for multiple testing. SPSS statistical software (version 25.0) was used.

## Results

### Study population

**Fig. 1:**
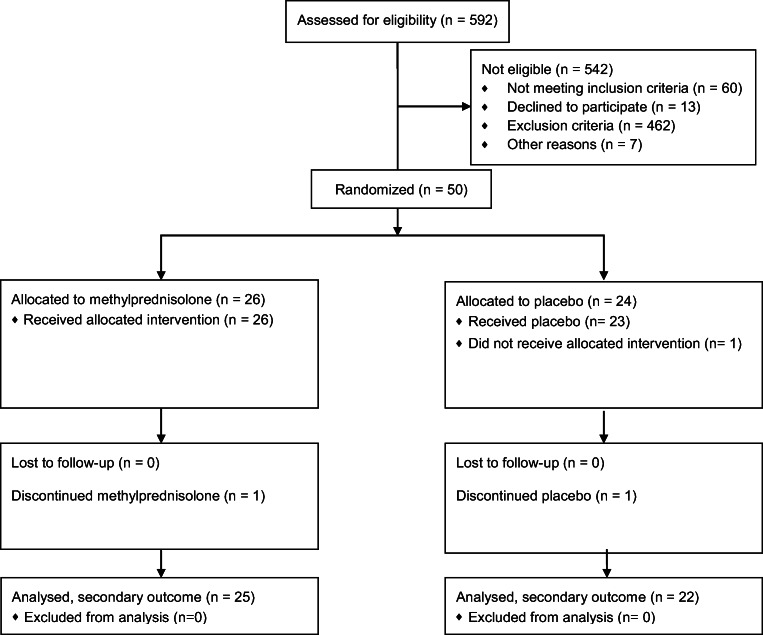
CONSORT flow diagram of the progress through the phases of the trial. The figure reports the essentials for analysis of sleep and is adapted from the diagram in the original trial [27]

Fifty patients were randomized to methylprednisolone 16 mg twice daily or placebo, 26 were allocated to the methylprednisolone group and 24 to the placebo group, of which 25 could be evaluated in the methylprednisolone group and 22 in the placebo group (Fig. 1). Figure 1 reports the essentials for analysis of sleep and is adapted from the diagram in the original trial [27].

The overall mean age of patients was 64.1 years (SD 10.1), mean Karnofsky performance status was 67.1 (SD 13.3), 51% were female, and the mean opioid dose was 223 mg (SD 223). Table 1 shows baseline demographics and clinical characteristics for each treatment group.

**Table 1:** Demographic and clinical characteristics at inclusion

Characteristics	Methylprednisolone (*n* = 25)	Placebo (*n* = 22)
Age (years), mean (SD)	62.5 (8.6)	66.0 (11.3)
Gender, *n* (%)		
Female	13 (52)	11 (50)
Male	12 (48)	11 (50)
Body mass index (kg/m^2^), mean (SD)	24.2 (5.8)	21.7 (3.4)
Cancer diagnosis, *n* (%)		
Breast	1 (4)	1 (5)
Prostate	2 (8)	3 (14)
Gastrointestinal	6 (24)	5 (23)
Lung	6 (24)	5 (23)
Gynecologic	5 (20)	5 (23)
Other	7 (28)	3 (14)
Cognitive function, (MMSE), mean	27.1	27.0
Performance score, (KPS), mean (SD)	66.6 (14.1)	65.6 (13.8)
Concomitant disease, *n* (%)		
Cardiac	4 (16)	4 (18)
Vascular	5 (19)	8 (36)
Lung	3 (12)	4 (18)
Opioid analgesics, mean (SD) oral morphine equivalents^a^	273.8 (256.8)	165.8 (163.9)
Mean number of other medications (SD)	4.0 (2.1)	4.5 (2.0)
Hypnotics^b^, *n* (%)	9 (35)	6 (25)
Antidepressants^c^, *n* (%)	4 (15)	4 (18)
Benzodiazepines^d^, *n* (%)	3 (12)	6 (25)

*SD* standard deviation, *MMSE* Mini-Mental State Examination [31], *KPS* Karnofsky Performance Score: 0 (dead) to 100 (normal activity) [32]

^a^mg per day

^b^Hypnotics, i.e., zopiclone, nitrazepam, alimemazine

^c^Antidepressants (citalopram, escitalopram, sertraline, mirtazapine)

^d^Benzodiazepines (oxazepam)

### Patient-reported sleep

**Fig. 2:**
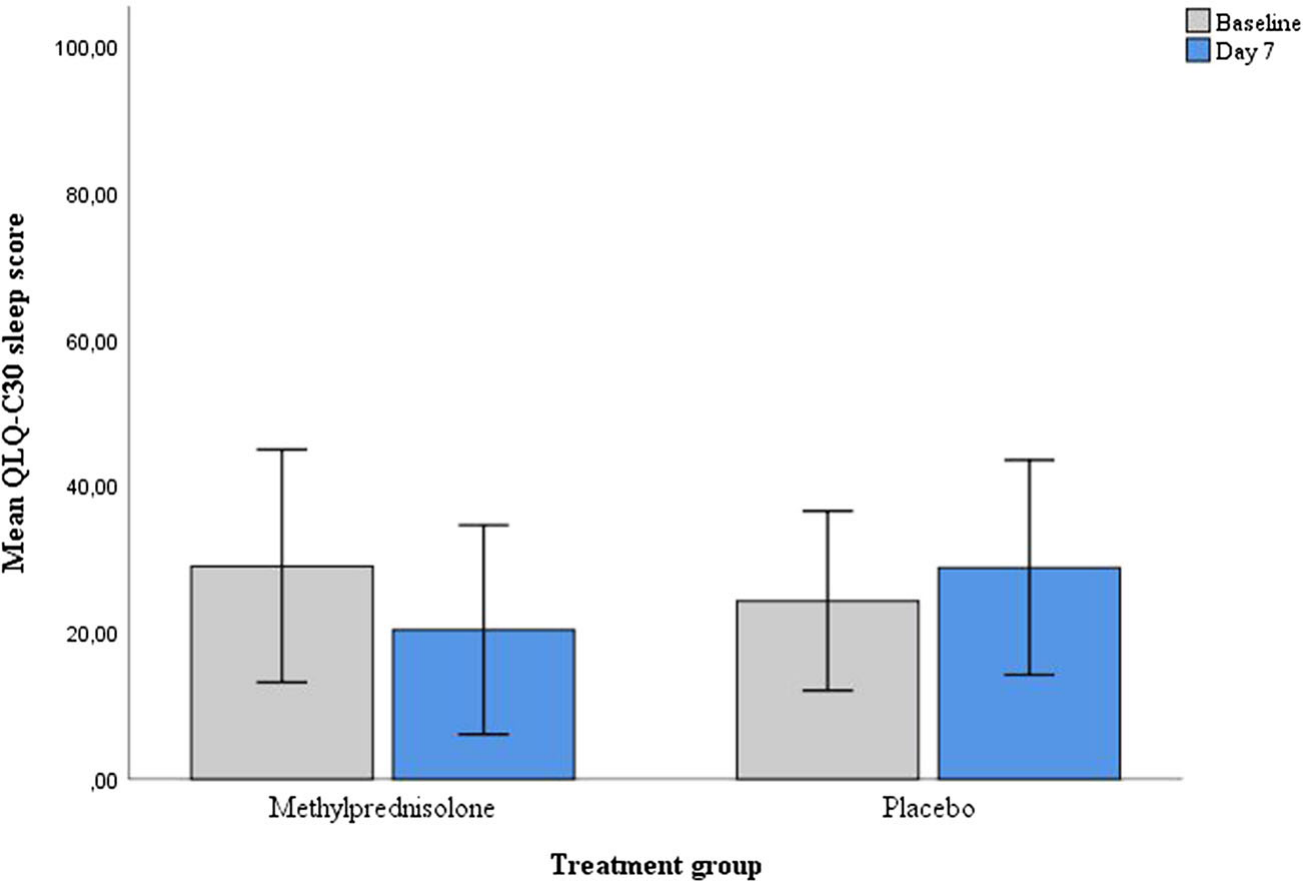
Mean score of QLQ-C30 sleep item according to treatment arm. A higher score represents more sleeping trouble. There were no statistically significant difference in change of sleep problems between the treatment arms (error bars 95% CI)

The mean QLQ-C30 sleep score at baseline was 29.0 (SD 36.7) in the methylprednisolone group (*n* = 23) and 24.2 (SD 27.6) in the placebo group (*n* = 22). At day 7, after controlling for pre-intervention scores on sleep, there were neither any clinical nor any statistically significant differences in the QLQ-C30 sleep score with methylprednisolone compared with placebo (ANCOVA, F = 1.92, *p* = 0.173) (Table 2; Fig. 2). The mean change in the QLQ-C30 sleep score was − 8.7 (SD 25.1) in the methylprednisolone group and 4.6 (SD 34.6) in the placebo group.

**Table 2:** Sleep scores at baseline and Day 7

Variable	Baseline Mean (SD)	Day 7 Mean (SD)	Mean change D0 to D7 (SD)	*p* value^a^
QLQ-C30 Sleep score^b^				
Methylprednisolone	29.0 (36.7)	20.3 (32.9)	− 8.7 (25.1)	0.173
Placebo	24.2 (27.6)	28.8 (33.0)	4.6 (34.6)	
PSQI, global score^c^				
Methylprednisolone	8.24 (4.25)	8.52 (5.02)	0.28 (2.01)	0.809
Placebo	7.64 (3.67)	8.05 (3.66)	0.41 (1.68)	
Subjective sleep quality^d^				
Methylprednisolone	1.08 (0.86)	0.92 (0.86)	− 0.16 (0.62)	0.305
Placebo	0.95 (0.79)	1.0 (0.82)	0.05 (0.58)	
Sleep latency^d^				
Methylprednisolone	1.20 (1.04)	1.24 (1.05)	0.04 (0.79)	0.217
Placebo	0.95 (0.90)	1.32 (0.84)	0.37 (0.66)	
Sleep duration^d^				
Methylprednisolone	0.48 (0.87)	0.60 (1.00)	0.12 (0.73)	0.994
Placebo	0.32 (0.78)	0.45 (0.85)	0.13 (0.35)	
Habitual sleep efficiency^d^				
Methylprednisolone	1.04 (1.10)	1.28 (1.21)	0.24 (0.78)	0.704
Placebo	0.82 (1.05)	1.00 (1.20)	0.18 (0.66)	
Sleep disturbance^d^				
Methylprednisolone	1.32 (0.63)	1.32 (0.48)	0.00 (0.71)	0.805
Placebo	1.36 (0.58)	1.36 (0.49)	0.00 (0.66)	
Use of sleep medication^d^				
Methylprednisolone	1.80 (1.44)	1.84 (1.46)	0.04 (0.20)	0.171
Placebo	1.73 (1.45)	1.68 (1.49)	− 0.05 (0.21)	
Daytime dysfunction^d^				
Methylprednisolone	1.32 (1.03)	1.32 (0.94)	0.00 (1.00)	0.414
Placebo	1.50 (0.96)	1.23 (0.87)	− 0.27 (0.90)	
Sleep onset latency, min^e^				
Methylprednisolone	23.7 (18.8)	22.5 (17.6)	− 1.1 (13.6)	0.118
Placebo	30.3 (39.1)	32.6 (36.4)	3.5 (11.6)	

^a^*p* value for comparison of follow up scores for the study groups (methylprednisolone and placebo) from the ANCOVA model

^b^EORTC QLQ-C30 symptom scale (0–100) where a high score denotes higher symptom burden [28]

^c^Pittsburg Sleep Quality Index global score 0–21, where a high score denotes more sleep problems [30]

^d^PSQI component score 0–3

^e^Sleep onset latency in minutes from the PSQI

At baseline, the mean PSQI global score was 8.2 (SD 4.3) and 7.6 (SD 3.7) in the methylprednisolone (*n* = 25) and placebo (*n* = 22) groups, respectively. At baseline, 18 of 25 (72%) of the patients in the methylprednisolone group and 15 of 22 (68%) in the placebo group were categorized as poor sleepers (PSQI global score > 5). At day 7, there were no significant differences between the groups in the global PSQI score (*p* = 0.809) or any of the single PSQI dimensions (Table 2).

## Discussion

The main finding of this trial was that treatment with methylprednisolone 16 mg twice daily for 7 days in patients with advanced cancer did not result in more patient-reported sleep problems as measured by the QLQ-C30 sleep score and the PSQI.

Our finding is similar to the results of a prospective, randomized, double-blind, placebo-controlled trial which primarily assessed the effects of oral dexamethasone 4 mg twice daily on cancer-related fatigue [26]. In that study, Yennurajalingam et al. found no change in sleep scores, as measured by a NRS of 0 to 10, in the dexamethasone group compared with the placebo group during 14 days of treatment in patients with advanced cancer. Our findings also agree with a cross-sectional study that observed no association between sleep disturbance and the use of corticosteroids in 442 advanced cancer patients in outpatient palliative care [34].

In contrast, other studies have demonstrated an association between use of corticosteroids and sleep disturbance in patients with advanced cancer. Mercadante et al. reported that use of corticosteroids was positively associated with subjective sleep disturbance [23] and was associated with significant sleep problems [19]. Moreover, insomnia was frequently reported in two prospective studies on corticosteroids for symptom control in palliative patients [24, 25]. However, these studies are limited by lack of a comparison group and not using a standardized dose of corticosteroids.

Several issues need to be addressed to interpret the findings from studies of sleep in palliative care. First, it may be difficult to differentiate corticosteroid adverse effects from symptoms related to a progressing malignant disease in patients with advanced cancer [24]. Compared to patients with less advanced disease, an isolated effect of corticosteroid use on sleep may be difficult to ascertain in patients with advanced disease who often have a higher symptom burden [35]. For instance, Chrousos et al. performed a placebo-controlled randomized trial and found that 50% of patients taking prednisolone for optic neuritis reported sleep disturbance, compared with 20% in the placebo group [36]. Additionally, corticosteroids may improve other symptoms in patients with advanced cancer which may indirectly influence sleep. In the present study, patient-reported appetite, fatigue, and patient satisfaction significantly improved in the methylprednisolone group [27]. Another example is a randomized controlled trial showing corticosteroids to be effective in improving cancer-related fatigue [26]. A qualitative study by Lundström et al. reported that corticosteroids administered for symptom control in patients with advanced metastatic cancer had positive existential consequences [37]. Moreover, studies have shown an association between the symptoms of sleep and fatigue [38, 39]. Thus, in patients with advanced cancer positive effects of corticosteroids on other symptoms that influence sleep may counter-balance potential direct negative short-term effects from corticosteroids on patient-reported sleep.

The second issue relates to the level of patient-reported sleep at baseline. In our trial, the QLQ-C30 sleep scores of 24 and 29 (0–100) in the study groups at baseline are in line with results from other studies in advanced-cancer patients [40–42], indicating poorer sleep compared with the general population [43]. Moreover, we found that 70% of the patients reported poor sleep quality on the PSQI before study start, echoing results from other studies with prevalence of poor sleep quality from 40 to 96% in patients with advanced cancer [2–5]. Thus, poor sleep quality at baseline in our trial might explain why these patients responded differently to corticosteroids than observed in other cohorts.

The third issue relates to the dose and timing of corticosteroids. The equipotent dose of methylprednisolone 32 mg in our trial equals about 6.4 mg dexamethasone, which was slightly lower than the dexamethasone dose of 8 mg in the randomized controlled trial by Yennurajalingam et al. [26]. The daily dose of corticosteroids is a risk factor for the development of sleep disturbance [15], i.e., higher doses of corticosteroids having more impact on sleep quality. Furthermore, the time of administration of corticosteroids may be an important factor for the impact on sleep. In our trial, patients were requested to take methylprednisolone in the morning and before 6 p.m. in the afternoon. Additionally, methylprednisolone has a shorter half-life compared with dexamethasone. This may have prevented sleep disturbance attributed to administration of corticosteroids late in the evening.

A final issue is the duration of the present trial. Since patients with advanced cancer are frail, attrition may make a longer study duration unfeasible [44]. On the other hand, a short study will not detect long-term effects. However, a review reported that 39–86% of adverse psychiatric effects, including insomnia, occurred during the first week after start of corticosteroids [15]. Thus, we consider a duration of 7 days to be adequate to explore the short-term impact of corticosteroids on patient-reported sleep in this cohort of patients with advanced cancer.

We recognize some limitations in this trial. First, patient-reported sleep was a secondary outcome in this randomized trial. Accordingly, the initial sample size estimation and power calculations were estimated for changes in self-reported pain, not sleep. Further, the sample size was small, which is reflected by the wide 95% confidence intervals. However, the mean QLQ-C30 sleep score actually improved in the methylprednisolone group compared with a deterioration in the placebo group, which supports the conclusion that a deterioration due to a corticosteroid induced adverse effect in this cohort is unlikely (i.e., a type II error). Second, the recall period of PSQI of 1 month makes it less appropriate for studies with shorter intervention periods. However, in the present trial, we could not detect an impact on sleep after 7 days of intervention neither with a questionnaire using a recall period of 1 week (QLQ-C30) nor with a recall period of 1 month (PSQI). Moreover, a study that examined the ecological validity for each of the items on the PSQI reported that patients’ results were comparable regardless of the length of the recall period from 3 days through a month [45]. Third, only a minor part of screened patients was eligible which leads to a risk for a selection bias. Finally, despite randomization, the corticosteroid group had slightly higher absolute levels of opioid consumption at baseline compared with the placebo group. Exploratory analysis including opioid consumption as a covariate did not change the results (data not reported). Thus, it is highly unlikely that the opioid consumption had any influence on the results.

This trial has several strengths. First, this was a randomized, placebo-controlled, double-blind trial using a standardized dosing of corticosteroids. Second, this trial recruited the predefined number of patients and had few dropouts compared with other trials in this patient group [26]. Third, the patient-reported outcomes are consistent with the previously published investigator report of adverse effects (AEs) in the present trial. The presence of adverse effects was assessed by the investigator through semi-structured interviews (presence of predefined AE category [yes vs. no] at day 7). “Sleeplessness” was noted by four participants in the methylprednisolone group as compared with three participants in the placebo group [27]. Finally, we consider these results important and relevant for clinical practice. The cohort studied represented patients with advanced cancer and a high symptom burden. As corticosteroids are commonly used, it is important to know that the use of methylprednisolone is well tolerated, also in terms of sleep.

## Conclusions

In conclusion, methylprednisolone 16 mg twice daily for 7 days had no impact on patient-reported sleep in a cohort of patients with advanced cancer treated with opioids. The majority of patients in this cohort reported poor sleep quality at baseline, emphasizing the need to address and treat sleep disturbances. The effects of long-term administration of corticosteroids on sleep in patients with advanced cancer need to be examined in a future study.

## Data Availability

The datasets generated and/or analyzed during the current study are available from the corresponding author on reasonable request.
